# The chloroplast genome of *Ostericum citriodorum* (Apiaceae), an endemic medicinal plant to China

**DOI:** 10.1080/23802359.2020.1778560

**Published:** 2020-11-20

**Authors:** Chenyang Liao, Qing Gao, Fen Wu

**Affiliations:** College of Architecture and Environment, Sichuan University, Chengdu, P. R. China

**Keywords:** Apiaceae, chloroplast genome, herbal medicine, *Ostericum citriodorum*

## Abstract

*Ostericum citriodorum* is a traditional Chinese medicinal herb endemic to Southeast and South China, but now is becoming very rare because of rapid habit loss. The complete chloroplast genome of *O. citriodorum* was sequenced herein and suggested that the complete chloroplast genome was 155,919 bp in length, comprising the large single-copy (LSC) region of 85,393 bp, the small single-copy (SSC) region of 19,760 bp, and a pair of inverted regions (IRs) of 25,383 bp. Totally 127 genes were distributed in the whole genome, including 4 rRNAs, 37 tRNAs, and 81 protein coding genes. The G + C content of this chloroplast genome was 38%. Phylogenetic inference revealed that *O. citriodorum* was accompanied with *Pterygopleurum neurophyllum* and sister to *O. palustre*, indicating a close relationship between *Ostericum* and *Pterygopleurum*.

*Ostericum citriodorum* (Hance) R.H. Shan & C.Q. Yuan is a striking perennial umbelliferous herb that has been used as a traditional Chinese medicine for treating angina pectoris (Shan 1992; Luo et al. 2020). As an endemic plant to China, this species is widespread in the hills or low mountains of Southeast and South China, but now is seriously threatened by rapid habit loss (Sheh et al. 2005; Liao et al. 2013). This research generated the first complete chloroplast genome of *O. citriodorum* aim to provide genomic information for the effective conservation and accurate identification of this species.

Fresh materials were obtained from the cultivated *O. citriodorum* in the green house of Sichuan University, Chengdu, China, which was transplanted from Luzhai, Guangxi, China. Both the voucher specimens and DNA samples were deposited in the herbarium of Sichuan University (accession no. lcy20120712). Morphological analysis was conducted by Karyotype (Altınordu et al. 2016). Total genomic DNA was extracted using Plant Genomic DNA Kit. The isolated genomic DNA was constructed to an average 400 bp paired-end (PE) library using the Illumina Hiseq platform (Illumina, San Diego, CA), and sequenced using Illumina genome analyzer (Hiseq PE150). The gene reconstruction, annotation, and analysis were carried out in Geneious Prime 2020.1.2 (Kearse et al. 2012), referring to *Pterygopleurum neurophyllum* (Maxim.) Kitag. (GenBank no. NC033345.1). The complete assemble genome sequence was deposited in the GenBank (accession no. MT501096).

The complete chloroplast genome of *O. citriodorum* was totally 155,919 bp in length with a circular form, and divided into four regions: the large single-copy region (LSC) of 85,393 bp, the small single-copy (SSC) region of 19,760 bp, and two inverted regions (IRs) of 25,383 bp each. The total GC content of this species was 38%, including 36% for the LSC, 31% for the SSC, and 43% for each IR. There were four rRNAs, 37 tRNAs, and 81 protein coding genes, for a total of 127 genes was annotated through the whole chloroplast genome. The tRNA coding genes were widely distributed in the genome, including 22 located in the LSC, 7 in each IR and only 1 in the SSC, while the rRNA coding genes exclusively placed in the IRs.

An alignment comprising the complete chloroplast genome sequences of *O. citriodorum* and other 17 related taxa of Apiaceae was performed using MAFFT (Katoh et al. 2002), and then trimmed and manually corrected using trimAl v1.4 (Capella-Gutierrez et al. 2009). Afterwards, neighbour-joining (NJ) trees were generated using MEGA7.0 (Kumar et al. 2016) with 1000 bootstrap replicates (Figure 1). The result suggested that *O. citriodorum* was accompanied with *Pterygopleurum neurophyllum* and sister to *O. palustre*, which was consistent with our previous research on morphology and molecular phylogeny of *Ostericum* (Liao et al. 2013; Li et al. 2017; Zhang et al. 2018; Liao et al. 2020), and also indicated a new point of view on the relationship between the genera of *Ostericum* and *Pterygopleurum*.

## Data Availability

All the chloroplast genome sequences used in this study are derived from the GenBank database at http://www.ncbi.nlm.nih.gov/genbank, and the reference numbers are DQ898156.1, GU456628.1, KU866530.1, KU921430.2, KX352486.1, MG719855.1, MH121055.1, MH180258.1, MN275034, MN539269.1, MN970215, MT501096, NC029392.1, NC029470.1, NC033343.1, NC033345.1, NC034645.1, NC042242.1, respectively. The neighbour-joining (NJ) analysis of *Ostericum citriodorum* and related taxa in Apiaceae, inferred from their complete chloroplast genome sequences. Bootstrap (BS) values from 1000 replicates are labeled on the nodes.
